# Editorial: Insights in General Cardiovascular Medicine: 2021

**DOI:** 10.3389/fcvm.2022.957636

**Published:** 2022-07-26

**Authors:** Lijun Wang, Jianyun Liu, Yi Lu, Maurizio Acampa, Pier Leopoldo Capecchi, Pietro Enea Lazzerini, Junjie Xiao

**Affiliations:** ^1^Affiliated Nantong Hospital of Shanghai University (The Sixth People's Hospital of Nantong), School of Medicine, Institute of Geriatrics (Shanghai University), Shanghai University, Nantong, China; ^2^Cardiac Regeneration and Ageing Lab, Shanghai Engineering Research Center of Organ Repair, School of Life Science, Institute of Cardiovascular Sciences, Shanghai University, Shanghai, China; ^3^Stroke Unit, Department of Emergency-Urgency and Transplants, “Santa Maria alle Scotte” General-Hospital, Azienda Ospedaliera Universitaria Senese, Siena, Italy; ^4^Department of Medical Sciences, Surgery and Neurosciences, University of Siena, Siena, Italy

**Keywords:** General Cardiovascular Medicine Section, Editorial, 2021, cardiovasccular medicine, cardiovascular disease

The goal of this Research Topic collection of articles published in *Frontiers in Cardiovascular Medicine: Insights in General Cardiovascular Medicine: 2021*, is to bring together excellent manuscripts of high-quality and high-citation potential, all of which gave been contributed by our editorial board members. Our criteria for selection include whether the topic addressed is focused on new discoveries and developments, current advances and challenges, as well as future prospects in the field of cardiovascular medicine research. This collection represents the quality and breadth of the papers published in this section, as well as the geographical variety of our scientific community. The rest of this editorial provides an overview of the articles included in the *Insights in General Cardiovascular Medicine: 2021* collection (Table 1).

**Table 1:** Metrics (on May 17, 2022) of the articles published in Insights in General Cardiovascular Medicine 2021.

**Title**	**First author, Country**	**Downloads**	**Views^**#**^**
The Post-thrombotic Syndrome-Prevention and Treatment: VAS-European Independent Foundation in Angiology/Vascular Medicine Position Paper	Benilde Cosmi, Italy	618	1,843
Diabetes and Its Cardiovascular Complications: Comprehensive Network and Systematic Analyses	Hao Wu, United States	273	1,711
EMbedding and Backscattered Scanning Electron Microscopy: A Detailed Protocol for the Whole-Specimen, High-Resolution Analysis of Cardiovascular Tissues	Rinat A. Mukhamadiyarov,Russia	489	1,575
Identification of Novel Single-Nucleotide Variants With Potential of Mediating Malfunction of MicroRNA in Congenital Heart Disease	Wangkai Liu, China	349	1,476
Screening for Asymptomatic Coronary Artery Disease via Exercise Stress Testing in Patients With Type 2 Diabetes Mellitus: A Systematic Review and Meta-Analysis	Yaoshan Dun, China	424	1,436
Relevance of Cor Pulmonale in COPD With and Without Pulmonary Hypertension: A Retrospective Cohort Study	Athiththan Yogeswaran, Germany	305	1,361
Cognitive Impairment in Heart Failure: Landscape, Challenges, and Future Directions	Mengxi Yang, China	269	1,342
Diagnostic and Therapeutic Management of the Thoracic Outlet Syndrome. Review of the Literature and Report of an Italian Experience	Giuseppe Camporese, Italy	194	1,141
Cardiorespiratory Responses During High-Intensity Interval Training Prescribed by Rating of Perceived Exertion in Patients After Myocardial Infarction Enrolled in Early Outpatient Cardiac Rehabilitation	Yaoshan Dun, China	371	1,127
Determination of Agrin and Related Proteins Levels as a Function of Age in Human Hearts	Katie L. Skeffington, United Kingdom	230	1,061
Vascular Stem/Progenitor Cells in Vessel Injury and Repair	Jiaping Tao, China	268	949
Rutaecarpine Inhibits Doxorubicin-Induced Oxidative Stress and Apoptosis by Activating AKT Signaling Pathway	Zi-Qi Liao, China	327	928
Valosin Containing Protein as a Specific Biomarker for Predicting the Development of Acute Coronary Syndrome and Its Complication	Chenchao Xu, China	153	915
NAP1L5 Promotes Nucleolar Hypertrophy and Is Required for Translation Activation During Cardiomyocyte Hypertrophy	Ningning Guo, China	275	857
Computed-Tomography as First-line Diagnostic Procedure in Patients With Out-of-Hospital Cardiac Arrest	John Adel, Germany	250	833
Association of Body Weight Variability With Progression of Coronary Artery Calcification in Patients With Predialysis Chronic Kidney Disease	Sang Heon Suh, South Korea	226	808
Clinical Characteristics for the Improvement of Cushing's Syndrome Complicated With Cardiomyopathy After Treatment With a Literature Review	Sisi Miao, China	250	761
Comparability of Heart Rate Turbulence Methodology: 15 Intervals Suffice to Calculate Turbulence Slope – A Methodological Analysis Using PhysioNet Data of 1,074 Patients	Valeria Blesius, Germany	91	691
Left Ventricular Strains and Myocardial Work in Adolescents With Anorexia Nervosa	Justine Paysal, France	158	686
A Novel Risk Score to Predict In-Hospital Mortality in Patients With Acute Myocardial Infarction: Results From a Prospective Observational Cohort	Lulu Li, China	119	591
Renal Denervation Attenuates Adverse Remodeling and Intramyocardial Inflammation in Acute Myocardial Infarction With Ischemia–Reperfusion Injury	Kun Wang, China	22	353

^#^*The order of appearance according to the number of views (on May 17, 2022)*.

## Insights in General Cardiovascular Medicine: 2021

The order of appearance according to the number of views (on May 17, 2022).

– *The Post-thrombotic Syndrome-Prevention and Treatment: VAS-European Independent Foundation in Angiology/Vascular Medicine Position Paper* (Cosmi et al.).

Post-thrombotic syndrome is a common and potentially risky complication of deep vein thrombosis that significantly affects patients' life quality. However, there are still great uncertainties in diagnosis, prevention, and treatment due to limited evidence-based approaches to clinical management. This position paper provided a practical framework and guidance for clinicians in PTS management.

– *Diabetes and Its Cardiovascular Complications: Comprehensive Network and Systematic Analyses* (Wu et al.).

There is currently no cure for diabetes. This review highlights current advances in the study of diabetes mechanisms using multi-omics analytical approaches and recent findings on the relationship between diabetes and other biological processes. This review provides important information for the prediction, diagnosis, and treatment of diabetes that could be useful for both clinicians and researchers.

– *EMbedding and Backscattered Scanning Electron Microscopy: A Detailed Protocol for the Whole-Specimen, High-Resolution Analysis of Cardiovascular Tissues* (Mukhamadiyarov et al.).

Analysis of cardiovascular tissue ultrastructure is complex. The current commonly used study methods have limitations such as low resolution and low signal-to-noise ratio. This protocol developed a new experimental method and provided a novel approach for imaging cardiovascular pathophysiological processes.

– *Identification of Novel Single-Nucleotide Variants With Potential of Mediating Malfunction of MicroRNA in Congenital Heart Disease* (Liu et al.).

Genetic mutation is the major cause of congenital heart defects (CHDs). This study identified miRNAs-mediated regulating on single-nucleotide variants from 3'-UTR of CHD-associated genes. This study suggests that miRNA-related gene regulation may be important but overlooked in the etiology of human congenital heart disease, suggesting that miRNA-related gene regulation should receive more attention in future CHD studies. The observations in this paper have important implications for clinicians.

– *Screening for Asymptomatic Coronary Artery Disease via Exercise Stress Testing in Patients With Type 2 Diabetes Mellitus: A Systematic Review and Meta-Analysis* (Dun, Wu et al.).

This systematic meta-analysis studied the exercise stress testing (ETS) screen program for asymptomatic cardiovascular diseases in type 2 diabetes mellitus, demonstrating its moderate sensitivity and specificity in the initial screening. ETS is a very promising tool due to the advantages of being non-invasive, relatively inexpensive, easily available in most centers, and the fact that it involves no radiation. Further additional studies are warranted to address the detailed flow and timing of ETS.

– *Relevance of Cor Pulmonale in COPD With and Without Pulmonary Hypertension: A Retrospective Cohort Study* (Yogeswaran et al.).

Pulmonary hypertension (PH), a complication of chronic obstructive pulmonary disease, is usually mild to moderate but can be severe in some patients. This paper suggests that cor pulmonale is associated with disease severity, providing recommendations for clinical researchers that cor pulmonale might be used to predict PH-COPD prognosis.

– *Cognitive Impairment in Heart Failure: Landscape, Challenges, and Future Directions* (Yang et al.).

This review summarizes research advances in the screening, diagnosis, and management of cognitive impairment (CI) in patients with heart failure, as well as the latest preventive therapies. The prevalence of CI in heart failure patients adds a greater burden on the patients' poor prognosis and worsening life quality. This paper provides important directions for clinicians and researchers that future research addresses the knowledge gaps in the field forward CI in heart failure.

– *Diagnostic and Therapeutic Management of the Thoracic Outlet Syndrome. Review of the Literature and Report of an Italian Experience* (Camporese et al.).

This paper reviews the diagnostic, therapy, and management of Thoracic Outlet Syndrome (TOS). A report in Italy was also analyzed. It provides recommendations for clinical researchers in the treatment of TOS.

– *Cardiorespiratory Responses During High-Intensity Interval Training Prescribed by Rating of Perceived Exertion in Patients After Myocardial Infarction Enrolled in Early Outpatient Cardiac Rehabilitation* (Dun, Hammer et al.).

Exercise training is an effective strategy to improve cardiorespiratory and benefits the cardiovascular system (1, 2). This study suggests that high-intensity interval training can be effectively prescribed using perceived exertion in myocardial infarction patients during early outpatient cardiac rehabilitation. This paper also provides information for researchers and clinicians to choose appropriate exercise regimes in cardiovascular medicine.

– *Determination of Agrin and Related Proteins Levels as a Function of Age in Human Hearts* (Skeffington et al.).

A variety of strategies have been studied to promote endogenous proliferation of cardiomyocytes and promote cardiac injury repair (3, 4). This study suggests that agrin was gradually decreased in human right ventricular tissue with aging but not in mice. This study shows the differences between rodents and humans, prompting researchers to pay attention to species differences in future investigations.

– *Vascular Stem/Progenitor Cells in Vessel Injury and Repair* (Tao et al.).

This review summarizes the latest research progress on Vascular Stem/Progenitor Cells in vessel injury and repair. Stem/progenitor cells play an important role in the regeneration and recruitment of damaged vascular cells during vascular repair. This paper discusses potential future research directions for stem cell therapy and provides important ideas for researchers in this field.

– *Rutaecarpine Inhibits Doxorubicin-Induced Oxidative Stress and Apoptosis by Activating AKT Signaling Pathway* (Liao et al.).

Cardiotoxicity is one of the main adverse reactions of doxorubicin in clinical cancer therapy applications (5, 6). This paper studies the protective effects of *Rutaecarpine* in doxorubicin-induced cardiotoxicity. This finding provides a new potential therapeutic option for the treatment of doxorubicin-induced cardiotoxicity.

– *Valosin Containing Protein as a Specific Biomarker for Predicting the Development of Acute Coronary Syndrome and Its Complication* (Xu et al.).

This paper analyzes human serum samples and found that serum valosin containing protein (VCP) levels could act as a biomarker to predict the development of acute coronary syndrome (ACS) and its complication, ventricular dysfunction. Biomarker identification is important for disease diagnosis and treatment. This study could provide important information for clinicians and researchers that VCP should be involved with the diagnosis and treatment of ACS.

– *NAP1L5 Promotes Nucleolar Hypertrophy and Is Required for Translation Activation During Cardiomyocyte Hypertrophy* (Guo et al.).

Cardiac hypertrophy is a compensatory response of the heart to increased hemodynamic stress under various pressures, and if not relieved, it gradually develops into heart failure. This study showed the new role of Nap1l5 in translation control during the progression of cardiac hypertrophy. This paper provides a new potential therapeutic target for the treatment of cardiac hypertrophy and heart failure.

– *Computed-Tomography as First-line Diagnostic Procedure in Patients With Out-of-Hospital Cardiac Arrest* (Adel et al.).

Coronary angiography is critical in patients with suspected causes of coronary arrest. This study reported that computed tomography scans can improve outcomes after out-of-hospital cardiac arrest (OHCA). This observation provides information for clinicians and investigators that CT should be routinely included in the diagnostic workup of OHCA regardless of the presence or absence of an ischemic ECG patterns.

– *Association of Body Weight Variability With Progression of Coronary Artery Calcification in Patients With Predialysis Chronic Kidney Disease* (Suh et al.).

This paper explores the clinical significance of bodyweight variability (BWV) in patients with predialysis chronic kidney disease. It found that high BWV is independently associated with the progression of coronary artery calcification. This study provides important evidence of a high association between BWV and coronary artery calcification, and should be of concern to clinicians.

– *Clinical Characteristics for the Improvement of Cushing's Syndrome Complicated With Cardiomyopathy After Treatment With a Literature Review* (Miao et al.).

Cushing's syndrome (CS) complicated with cardiomyopathy is a rare clinical type with high mortality. This study retrospectively reviewed case reports from literature and demonstrated that cortisol played an important role, and hypercortisolemia can improve significantly after remission. This is an interesting finding, but the detailed mechanism needs further study.

– *Comparability of Heart Rate Turbulence Methodology: 15 Intervals Suffice to Calculate Turbulence Slope – A Methodological Analysis Using PhysioNet Data of 1074 Patients* (Blesius et al.).

This study focused on common variations in the number of intervals after ventricular premature contraction (VPC) that are used to calculate turbulence slope (TS). Heart rate turbulence (HRT) is a characteristic heart rate pattern triggered by a VPC. This paper provides important information for clinicians that HRT occurred at early intervals after the VPC, whereas TS calculated from a later time interval reflected overall heart rate variability rather than differential responses to VPC.

– *Left Ventricular Strains and Myocardial Work in Adolescents With Anorexia Nervosa* (Paysal et al.).

Cardiovascular disease is a common complication of anorexia nervosa (AN). This study suggests that the cardiac function in AN patients was preserved. The paper outlines that global longitudinal strain was higher in AN patients, while the clinical significance required further investigation.

– *A Novel Risk Score to Predict In-Hospital Mortality in Patients With Acute Myocardial Infarction: Results From a Prospective Observational Cohort* (Li et al.).

This study developed a novel HAMIOT (Heart Failure after Acute Myocardial Infarction with Optimal Treatment) risk score to predict in-hospital mortality of MI patients using 10 highly predictive variables. Using this tool, the risk score is easily calculated and the variables are easily obtained during hospitalization, offering a useful method for clinicians to predict the risk of mortality.

– *Renal Denervation Attenuates Adverse Remodeling and Intramyocardial Inflammation in Acute Myocardial Infarction With Ischemia–Reperfusion Injury* (Wang et al.).

Acute myocardial infarction (AMI) is the most severe symptom of coronary artery disease and the leading cause of morbidity and mortality worldwide. This paper studied the protective effects on renal denervation (RDN) in myocardial ischemia/reperfusion (I/R) pigs. This data provides compelling evidence for the applicability and efficacy of RDN and suggests that this treatment strategy needs further development.

## Author Contributions

JX, PL, PC, and MA are topic editors of this special issue and contributed to writing and revising of this editorial. LW, JL, and YL drafted the editorial. All authors contributed to the article and approved the submitted version.

## Funding

This work was supported by the grants from National Key Research and Development Project (2018YFE0113500 to JX), National Natural Science Foundation of China (82020108002 to JX), Innovation Program of Shanghai Municipal Education Commission (2017-01-07-00-09-E00042 to JX), the grant from Science and Technology Commission of Shanghai Municipality (21XD1421300 and 20DZ2255400 to JX), Natural Science Foundation of Shanghai (19ZR1474100 to LW), and the “Dawn” Program of Shanghai Education Commission (19SG34 to JX).

## Conflict of Interest

The authors declare that the research was conducted in the absence of any commercial or financial relationships that could be construed as a potential conflict of interest.

## Publisher's Note

All claims expressed in this article are solely those of the authors and do not necessarily represent those of their affiliated organizations, or those of the publisher, the editors and the reviewers. Any product that may be evaluated in this article, or claim that may be made by its manufacturer, is not guaranteed or endorsed by the publisher.

## References

[1] Wang L Wang J Cretoiu D Li G Xiao J. Exercise-mediated regulation of autophagy in the cardiovascular system. J Sport Health Sci. (2020) 9:203–10. 10.1016/j.jshs.2019.10.001 32444145 PMC7242217

[2] Bernardo BC Ooi JYY Weeks KL Patterson NL McMullen JR. Understanding key mechanisms of exercise-induced cardiac protection to mitigate disease: current knowledge and emerging concepts. Physiol Rev. (2018) 98:419–75. 10.1152/physrev.00043.2016 29351515

[3] Wu X Wang L Wang K Li J Chen R Wu X. ADAR2 increases in exercised heart and protects against myocardial infarction and doxorubicin-induced cardiotoxicity. Mol Ther. (2022) 30:400–14. 10.1016/j.ymthe.2021.07.004 34274534 PMC8753375

[4] Vujic A Lerchenmuller C Wu TD Guillermier C Rabolli CP Gonzalez E. Exercise induces new cardiomyocyte generation in the adult mammalian heart. Nat Commun. (2018) 9:1659. 10.1038/s41467-018-04083-1 29695718 PMC5916892

[5] Bao YW Hua XW Zeng J Wu FG. Bacterial template synthesis of multifunctional nanospindles for glutathione detection and enhanced cancer-specific chemo-chemodynamic therapy. Research (Wash D C). (2020) 2020:9301215. 10.34133/2020/9301215 32529190 PMC7136754

[6] Rochette L Guenancia C Gudjoncik A Hachet O Zeller M Cottin Y. Anthracyclines/trastuzumab: new aspects of cardiotoxicity and molecular mechanisms. Trends Pharmacol Sci. (2015) 36:326–48. 10.1016/j.tips.2015.03.005 25895646

